# Foot Length, Chest Circumference, and Mid Upper Arm Circumference Are Good Predictors of Low Birth Weight and Prematurity in Ethnic Minority Newborns in Vietnam: A Hospital-Based Observational Study

**DOI:** 10.1371/journal.pone.0142420

**Published:** 2015-11-10

**Authors:** Hai Nguyen Thi, Dung Khu Thi Khanh, Ha Le Thi Thu, Emma G. Thomas, Katherine J. Lee, Fiona M. Russell

**Affiliations:** 1 Department of Pediatrics, Hoa Binh General Hospital, Hoa Binh, Vietnam; 2 Neonatal Department, National Hospital of Pediatrics, Hanoi, Vietnam; 3 Murdoch Childrens Research Institute, Melbourne, Parkville, Australia; 4 Department of Paediatrics, The University of Melbourne, Parkville, Australia; 5 Centre for International Child Health, Department of Paediatrics, The University of Melbourne, Parkville, Australia; 6 Murdoch Childrens Research Institute, Parkville, Australia; Institute of Preventive Medicine, DENMARK

## Abstract

**Background:**

The evaluation of tools to accurately identify low birth weight (LBW) and/or premature newborns in resource-limited countries is a research priority. We explored the use of foot length, chest circumference, and mid-upper arm circumference (MUAC) measured within 24 h as diagnostic tools for identifying newborns who are LBW, premature, or both; and compared measurements taken at birth with those taken at five days of age.

**Materials and Methods:**

An observational study was undertaken in Hoa Binh Province General Hospital, Vietnam, in ethnic minority newborns. Birth weight, foot length, chest circumference, and MUAC were measured within 24 h of birth and in a subset of 200, were repeated on day five of life. Gestational age was estimated using the New Ballard Score. Receiver Operating Characteristic curves and optimal cut-points (the point with the highest sensitivity and specificity where the sensitivity was at least 0.8) were calculated, for predicting prematurity, LBW, and both. Measurements within 24 h and at five days of life were compared.

**Results:**

485 newborns were recruited. Chest circumference and MUAC measured within 24 h of birth were found to be highly predictive of LBW (both yielding area under the curve [AUC] of 0.98, 95% confidence interval [CI] 0.96–0.99), and performed marginally better than foot length (AUC 0.94, 95%CI 0.92–0.96). The optimal cut-points for measurements within 24 h of birth were ≤7.4cm for foot length; ≤30.4cm for chest circumference; and ≤ 9.0cm for MUAC. There was statistical evidence that anthropometric measurements taken within 24 h of birth were higher than measurements on day five (p<0.02 for all anthropometric measurements) but the magnitude of these differences was small (at most 2mm).

**Conclusions:**

All measurements taken within 24 h of birth were good predictors of LBW, prematurity and both. Differences in measurements taken within 24 h and on day five were not clinically relevant. Further research will ensure that the application of these measures is reliable in community settings.

## Introduction

Low birth weight (LBW), prematurity and intrauterine growth retardation are very strong predictors of neonatal mortality [1]. In fact, preterm birth is the leading cause of child deaths worldwide [2], with 28% of all neonatal deaths estimated to be due to prematurity [3,4]. A large number of premature and LBW newborns who die during the neonatal period are moderately preterm. Low-cost and high impact interventions are recommended to improve the outcomes for these newborns [5]. However, as many such births occur in settings where accurate weighing scales or measures of gestational age are not available, there are currently no mechanisms to identify these small (premature and/or LBW) newborns in need of extra care. The identification and evaluation of low-cost tools to accurately identify small newborns in primary health care and community settings has been ranked as the number one research priority to reduce global mortality from prematurity and LBW [6].

There has been considerable interest in using simple anthropometric measures as a proxy for birth weight [5] to identify small newborns. Several studies in a variety of settings have shown that different anthropometric measurements at birth are highly predictive of birth weight and can be used as valid predictors of LBW [7]. A meta-analysis found chest circumference was the best anthropometric measurement to predict LBW [8]. However there are limitations in applying chest circumference measurements in the community as the newborn needs to be undressed, which may expose these small newborns to hypothermia, and obtaining an accurate measurement without medical training may be difficult [9,10]. Using alternative anthropometric measurements such as mid-upper arm circumference (MUAC) or foot length, which are less invasive, could be useful tools for identifying babies at high risk of mortality in resource-limited settings [6]. However, local validation is required to identify potential ethnic variations in anthropometric measurements.

Vietnam is a lower-middle income country which has shown substantial reductions in child mortality over the last 20 years [11]. However ethnic minority newborns have an almost three-fold higher risk of neonatal mortality compared with their Kinh (majority) counterparts [12]. In addition, previous studies have shown that as many as half of all women in remote and mountainous areas in Vietnam, where many ethnic minority women live, deliver their babies at home [13]. Shortage of equipment, drugs and staff in the health facilities [14], and poverty impede access to health care in this population [15]. In addition, many health facilities in rural areas, where most of the ethnic minority population resides, do not have accurate weighing scales or mechanisms to measure birth weight or gestational age (GA).

Identifying a simple tool to identify premature and LBW neonates born in settings without weighing scales and the availability of reliable GA assessments is urgently required. This is particularly important in places like Hoa Binh Province, Vietnam, where 73% of the population are from an ethnic minority group, and ~86% of the population live in rural and mountainous areas and do not have access to reliable GA assessments. The aims of this study in ethnic minority newborns in Vietnam are to: 1) explore the use of foot length, chest circumference, and MUAC taken within 24 h of birth as diagnostic tools for identifying small newborns who are LBW, are premature, or are both premature and LBW; and 2) compare anthropometric measures taken within 24 h with those taken at five days of age.

## Materials and Methods

### Study design

This was a hospital-based observational study of 485 newborns, with follow up on day five for the first 200 participants.

### Study setting

The study was undertaken in the Maternity and Neonatal departments of Hoa Binh Province General Hospital, Vietnam from 1^st^ June 2013 to 31^st^ August 2014. This hospital is located in north-western Vietnam, and serves a population of approximately 800,000. The biggest ethnic minority group in Hoa Binh Province are the Muong (63.4%), followed by Kinh (27.7%), Thai (3.9%), Tay (2.7%), Dao, and H’Mong [16]. There are approximately 3,000 births in this hospital each year, with about 15–20% of all deliveries being to ethnic minority mothers.

### Participants and study procedures

Ethics approval was attained from the National Hospital of Pediatrics Ethics Review Committee, Hanoi and the Human Research Ethics Committee, The University of Melbourne, which approved all study procedures, including the informed consent process, prior to study commencement. All study staff were trained by the pediatrician (FMR) in all study procedures prior to study commencement.

Ethnic minority (Tay, Dao, Nung, H’Mong, Muong, or San Chi) newborns <24 h old, whose parent/guardian consented to for them to participate in the study, were eligible to enrol. Newborns who had clinical signs of severe illness (including severe respiratory distress, birth asphyxia, seizures) were excluded.

During the study period, and within routine office hours, all babies born in the hospital were screened for inclusion. Study staff approached parents following the birth of their child, and were explained the study in their local language. Consent materials were translated to the local language. Following written informed consent (or for those illiterate, a witnessed thumb print was provided), a data collection form was completed by study staff by interview with the parent/guardian and a review of the medical records. The date of the last menstrual period (LMP) was sourced from the mother to estimate expected date of delivery and GA of the newborn. Medical records were reviewed for early ultrasound findings and estimated date of delivery. If this were not recorded, the information was requested from the mother.

Anthropometric measurements (weight, chest circumference, MUAC, and foot length) were taken immediately following recruitment by the trained pediatrician and recorded on the study specific data collections forms. All measurements were taken with the newborn naked. Newborns were weighed using a calibrated digital weighing scale (Salter® digital baby/toddler scales, model WS034) which allows readings to the nearest of 10 g. The scale was periodically calibrated using a set of standard 500 g weights. Chest circumference and MUAC measurements were recorded to the nearest 0.1 cm, using a non-elastic, flexible measuring tape. Chest circumference was measured at the level of the nipple during expiration. MUAC was measured at the mid-point between the tip of acromion process and olecranon process of the right upper arm. The right foot was measured from the center of the heel pad to the tip of the hallux (big) toe using a stiff transparent plastic metric ruler (Staedler®). All anthropometric measures were repeated three times, with the mean of the three repeat measurements used for analysis.

GA was clinically assessed using the New Ballard Score (NBS) assessment by a trained study pediatrician, since GA according to date of LMP and antenatal ultrasound were not available for all participants. The NBS comprises thirteen individual items, each of which classifies the physical and neurological characteristics of the newborn on a different ordinal scale (typically ranging between -1 and 4) [17]. The total NBS is the sum of the individual items and has a range of -10 to 50. GA was estimated from the NBS using the standard scoring guide [17]. As this guide only provides GA corresponding to NBS scores in five point increments, GA for the intermediate scores were obtained via linear interpolation between these increments using the following formula: GA = (2 × *NBS* + 120)/5 [18].

The NBS estimate of GA was dichotomised into premature (<37 weeks at birth) and term-born (≥37 weeks). Birth weight was dichotomised into LBW (<2500g) and normal birth weight (≥2500g). These binary measures formed the main outcomes of interest. For descriptive purposes, very premature (<32 weeks) and very LBW (<1500g) newborns were also identified.

Mothers who were able to return with their infant on day 5 for review, had their infants remeasured. Chest circumference, MUAC, foot length were remeasured by the study pediatrician, using the same approach as described above.

The study was carried out according to Good Clinical Practice. Standard operating procedures were developed for all study procedures. Anthropometric measurements and NBS were taken by two study pediatricians following training and supervision by the pediatrician (FMR). NHT was trained on the neonatal ward for five days prior to data collection, and anthropometric measurements and New Ballard scores were compared between NHT and FMR to assess accuracy. Clinical assessments were viewed by FMR during site visits. A second local pediatrician checked all data collection forms for logical errors and completeness prior to data entry. There was double data entry on 100% of all data collection forms. Only one set of digital weighing scales were used. The scales were checked with monthly standard calibration weights to ensure they were weighing accurately.

### Sample size

The sample size (n = 485) was obtained based on the number of newborns necessary to estimate the sensitivity of an anthropometric measurement as a diagnostic tool for identifying LBW newborns. Assuming 80% sensitivity, the minimum sensitivity thought to be acceptable for a diagnostic tool in this context, 97 LBW newborns would be required to estimate the sensitivity to within 8% (based on a 95% confidence interval). Assuming 17% prevalence of LBW, this equates to a total sample size of 485 newborns. Anthropometric measurements were retaken on day five of life in those who were able to return for review.

### Statistical analyses

The characteristics of the study participants are described using means and standard deviations for continuous variables, or the median and interquartile range for skewed data, and the number and percentage in each category for categorical variables. Two sample t-tests were undertaken to test for sex differences in GA and birth weight. The most reliable measure of GA is expected date of delivery derived from dates calculated from an ultrasound in early pregnancy. However few mothers had an early ultrasound so instead we used NBS to estimate GA. To justify the use of the NBS for measuring GA, the correlation between GA measured according to the NBS, ultrasound and LMP is presented for those with data available.

To address aim 1, Receiver Operating Characteristic (ROC) curves are presented for the use of foot length, chest circumference and MUAC measured within 24 h of birth as diagnostic tools for each of three outcomes: prematurity, LBW, and being both premature and LBW. For each curve, the optimal cut-point was obtained, defined as the point with the highest sensitivity and specificity such that the sensitivity was at least 0.8. In other words, the optimal cut-point was chosen to minimise the distance from the ROC curve to the point (0,1), subject to the constraint that the sensitivity must be at least 0.8. The sensitivity, specificity, positive predictive value (PPV) and negative predictive value (NPV) at each of the chosen cut-points were computed along with 95% confidence intervals (CIs). We compared the sensitivity and specificity of different anthropometric measures using McNemar tests for a difference in paired proportions.

To address aim 2, scatterplots of each anthropometric measure taken within 24 h of birth and the corresponding measurement on day five of life are presented along with the correlation between the two measures. The mean difference in each pair of measurements was then estimated along with 95% CIs, with the null hypothesis of no difference between the two measures tested using paired t-tests.

Analyses were performed in Stata version 13.1.

## Results

**Fig 1:**
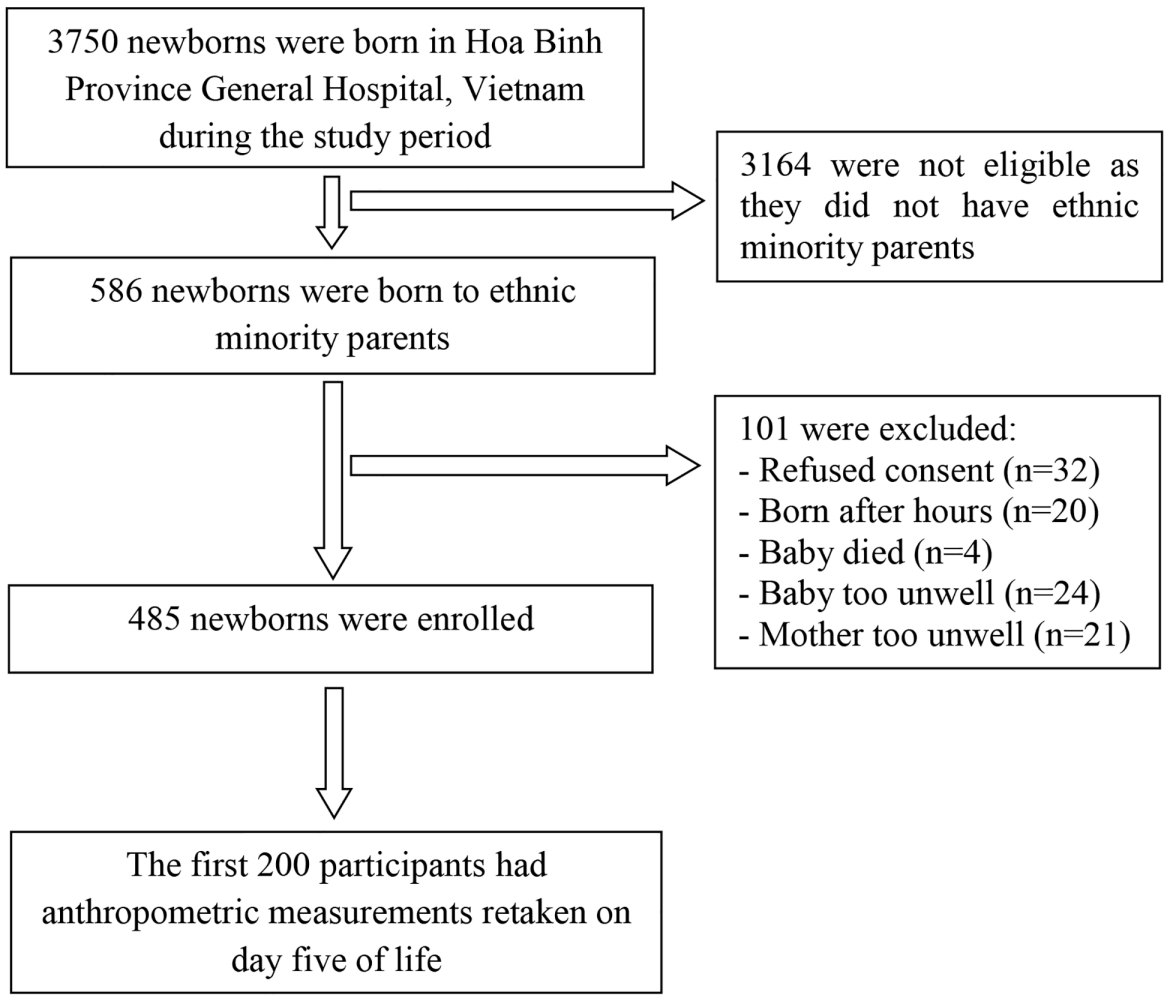
Flowchart of recruitment of study participants at Hoa Binh Province General Hospital, Vietnam.

Fig 1 shows the flowchart of recruitment of participants into the study. There were 485 newborns recruited into the study, comprising 82.8% of all ethnic minority deliveries during the study period. Table 1 summarises the demographic, physical and clinical characteristics of the study participants. The mean birth weight of all newborns was 2489 g, with 51% being LBW and 47% premature. Most preterm newborns (93%) were LBW, and few term newborns (11%) were LBW. Male newborns were estimated to be on average 98 g (95%CI -6 g to 200 g, p = 0.06) heavier and 0.08 weeks (95%CI -0.4 weeks to 0.5 weeks, p = 0.72) older in GA (according to the NBS) than their female peers. In the subset of 282 babies for whom date of LMP was available, there was good correlation (0.81) between GA by NBS and by LMP. In the subset of 391 babies for whom ultrasound information was available, the correlation between GA by NBS and by ultrasound was also high (0.90).

**Table 1:** Demographic and clinical characteristics of the study participants (N = 485).

Variable (N = 485, unless otherwise stated)	Summary	
Demographics	Number	Percent
Female	236	49
Ethnicity		
Muong	452	93
Tay	15	3
Dao	11	2
Other minority group	6	1
Gestational age^a^	Range	Median (interquartile range)
Gestational age in weeks by NBS	30.0–41.6	37.2 (35.0–38.6)
Gestational age in weeks by ultrasound (N = 391)	30.0–42.0	38 (35.0–39.0)
Gestational age in weeks by LMP (N = 282)	30.0–43.0	37 (35.0–39.0)
Gestational age groups by NBS	Number	Percent
Very premature (<32 weeks)	11	2
Premature (<37 weeks)	226	47
Birth weight	Range	Mean (standard deviation)
Birth weight (g)	1007–4500	2489 (58)
Birth weight categories	Number	Percent
Very LBW (<1500g)	21	4
LBW (<2500g)	246	51
Gestational age and birth weight categories	Number	Percent
Premature and low birth weight	193	40
Premature and normal birth weight	33	7
Term born and low birth weight	53	11
Anthropometric measures (day 1 of life)	Range	Mean (standard deviation)
Foot length (cm)	5.5–8.7	7.4 (0.6)
Chest circumference (cm)	25.8–36.6	30.4 (2.8)
Mid-upper arm circumference (cm)	22.6–40.5	8.9 (1.1)
Head circumference (cm)	6.3–11.0	32.4 (2.0)

^a^Medians are presented for measures of gestational age due to skewness of these variables. NBS = New Ballard Score

LMP = last menstrual period

Prematurity is defined as <37 weeks gestational age according to the NBS

LBW = Low birth weight, defined as <2500 g.

**Fig 2:**
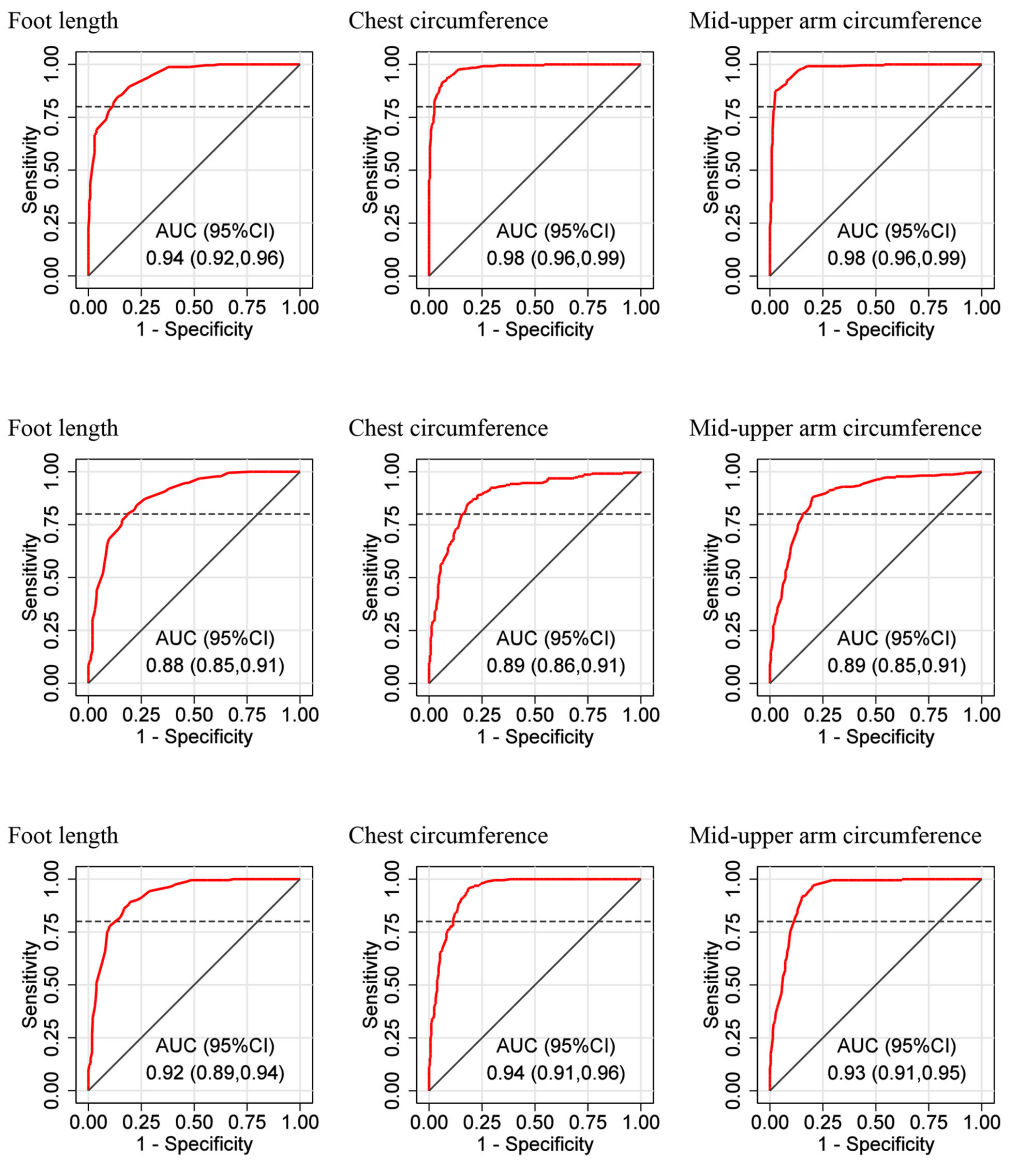
Receiver operating characteristic (ROC) curves for anthropometric measures as diagnostic tools for predicting each outcome, and areas under each curve (AUC). ***Top row*: ROC curves for anthropometric measures as predictors of low birth weight (<2500g). *Middle row*: ROC curves for anthropometric measures as predictors of prematurity (<37 weeks according to the New Ballard Score). *Bottom row*: ROC curves for anthropometric measures as predictors of low birth weight and prematurity.** The solid diagonal line represents a theoretical ROC curve that is no better than random as a predictor of the outcome; the dashed horizontal line represents the required threshold sensitivity of 0.8.

Fig 2 shows the ROC curves and AUCs for each anthropometric measure (foot length, chest circumference and MUAC) as a diagnostic tool for predicting each outcome (LBW, prematurity, and being both LBW and premature). Point estimates for the AUCs ranged from 0.88 to 0.98, suggesting that, overall, the anthropometric measures are strong predictors of all three outcomes. In general, the diagnostic performance of the anthropometric measures was best when LBW was the outcome (this outcome had the highest estimated AUCs), followed by both prematurity and LBW, and then prematurity.

Chest circumference and MUAC were very good predictors of LBW (both yielded estimated AUCs of 0.98, 95%CI 0.96 to 0.99), and performed marginally better than foot length (AUC 0.94, 95%CI 0.92 to 0.96). Diagnostic performance when predicting prematurity and both LBW and prematurity was similar among the anthropometric measures. Of note, there was substantial overlap between the 95% CIs for the AUCs across the anthropometric measures.

Table 2 presents the optimal cut-points for each anthropometric measure as a predictor of each outcome, along with the sensitivity, specificity, PPV and NPV based on the given cut-point. The optimal cut-points identified for each diagnostic tool were similar across the three outcomes: for foot length, these cut points were between 7.3 cm and 7.4 cm; for chest circumference, between 30.0 cm and 30.4 cm; for MUAC, between 8.7 cm and 9.0 cm.

**Table 2:** Sensitivity, specificity, and positive and negative predictive values of optimal cut-points^1^ for each outcome and anthropometric measure.

	Sensitivity (95%CI)	Specificity (95%CI)	PPV (95%CI)	NPV (95%CI)
Low birth weight (<2500g)				
Foot length ≤ 7.4cm	0.85 (0.79,0.89)	0.86 (0.81,0.90)	0.86 (0.81,0.90)	0.84 (0.79,0.89)
Chest circumference ≤ 30.4cm	0.91 (0.87,0.95)	0.94 (0.90,0.96)	0.94 (0.90,0.96)	0.91 (0.87,0.95)
MUAC^c^ ≤ 8.9cm	0.92 (0.88,0.95)	0.92 (0.88,0.95)	0.92 (0.88,0.95)	0.92 (0.88.0.95)
Pre-term (by New Ballard Score)				
Foot length ≤ 7.3cm	0.80 (0.74,0.85)	0.81 (0.76,0.86)	0.79 (0.73,0.84)	0.82 (0.77,0.87)
Chest circumference ≤ 30.4cm	0.85 (0.79,0.89)	0.82 (0.77,0.86)	0.80 (0.75,0.85)	0.86 (0.81,0.90)
MUAC^c^ ≤ 9.0cm	0.88 (0.83,0.92)	0.80 (0.75,0.85)	0.79 (0.74,0.84)	0.88 (0.84,0.92)
Low birth weight and pre-term				
Foot length ≤ 7.3cm	0.86 (0.80,0.91)	0.83 (0.78,0.87)	0.77 (0.71,0.83)	0.90 (0.86,0.93)
Chest circumference ≤ 30.0cm	0.91(0.86,0.95)	0.85 (0.80,0.89)	0.80 (0.74,0.85)	0.94 (0.90,0.96)
MUAC^c^ ≤ 8.7cm	0.92 (0.87,0.95)	0.85 (0.80,0.89)	0.80 (0.74,0.85)	0.94 (0.90,0.96)

^1^ The optimal cut-point was defined as the point on the ROC curve with a sensitivity ≥0.8 that minimised the distance from the ROC curve to the point (0,1).

PPV = positive predictive value

NPV = negative predictive value

MUAC = Mid-upper arm circumference.

The sensitivity and specificity at the optimal cut-points were similar for chest circumference and MUAC as predictors of each outcome (p for difference >0.05 in all cases), and were typically marginally higher than for foot length, with the strongest statistical evidence for such differences when LBW was the outcome (p<0.05 for all differences when LBW was the outcome). Since the optimal cut-points were chosen to have sensitivity of at least 0.80, by definition the point estimates of the sensitivity were always ≥0.80, but the 95%CIs sometimes included values below 0.80 (this occurred for foot length and MUAC as predictors of prematurity, and foot length as a predictor of LBW). Point estimates of the specificity at each optimal cut-point were also good (≥0.80) for all predictors of all three outcomes. Sensitivity and specificity at the optimal cut-points were generally highest for each diagnostic tool when predicting LBW.

**Fig 3:**
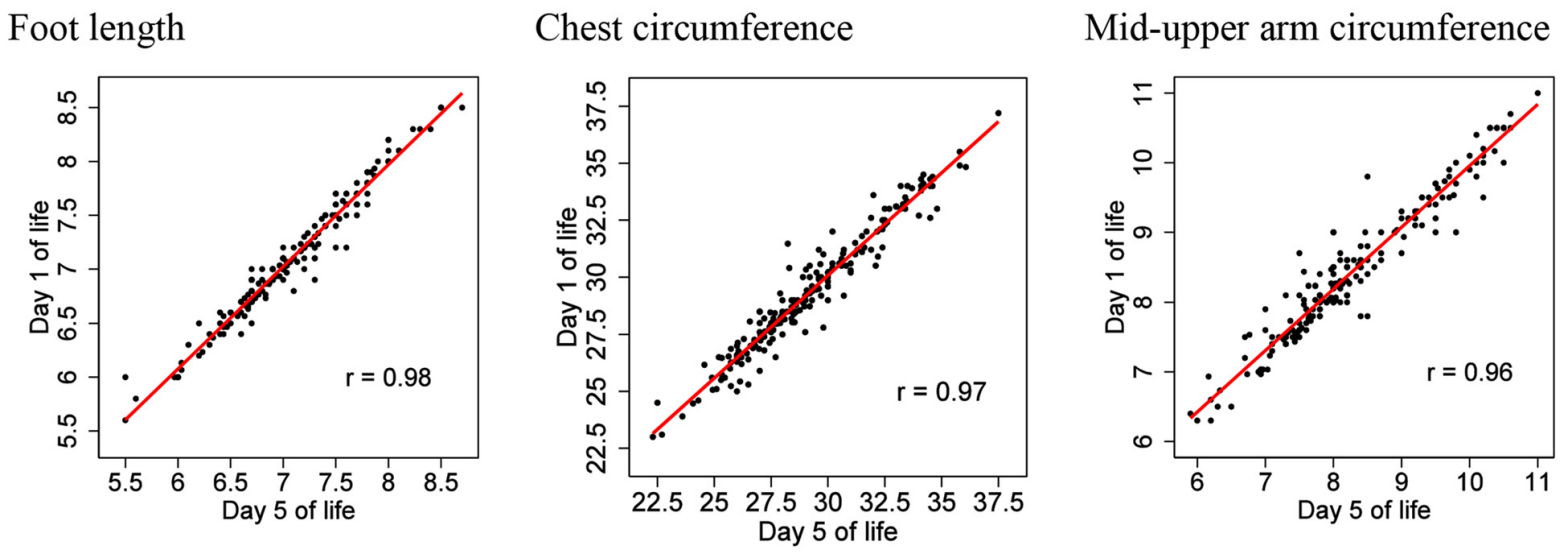
Scatter plots of anthropometric measurements taken at day 1 of life against measurements taken at day 5 of life (n = 200). The line of best fit and the correlation coefficient (*r)* are also shown. All measurements are in centimetres.

Fig 3 shows scatter plots of the anthropometric measurements taken within 24 h of birth against the same measurements at day five of life, along with lines of best fit and correlation coefficients (*r*) for these relationships, in the subset of 200 babies who were assessed at both time points. The pairs of measurements taken at each time point were highly correlated (*r* = 0.98 for foot length, *r* = 0.97 for chest circumference, and *r* = 0.96 for MUAC).

Table 3 shows the mean of foot length, chest circumference and MUAC taken within 24 h of birth and day five of life, and the estimated mean difference between pairs of measurements, in the subset of newborns who were assessed at both time points (N = 200). On average, the measurements were slightly lower at day five compared with those taken within 24 h of birth (foot length: mean decrease 0.018 cm, 95%CI 0.003 to 0.033; chest circumference: 0.162 cm, 95%CI 0.062 to 0.262; MUAC: 0.157 cm, 95%CI 0.117 to 0.198). However, the estimated differences were small (<0.2 cm in all cases).

**Table 3:** Comparison of anthropometric measures at days 1 and 5 of life, in a subset of newborns remeasured on day 5 (N = 200).

Anthropometric measure	Mean (SD) at day 1 of life	Mean (SD) at day 5 of life	Mean difference (day 1 –day 5) (95%CI)	p-value
Foot length (cm)	7.13 (0.55)	7.11 (0.57)	0.018 (0.003,0.033)	0.0184
Chest circumference (cm)	29.32 (2.70)	29.16 (2.91)	0.162 (0.062,0.262)	0.0017
Mid-upper arm circumference (cm)	8.43 (0.99)	8.28 (1.08)	0.157 (0.117,0.198)	<0.0001

## Discussion

In this study of ethnicity minority newborns in rural Vietnam, we found that chest circumference, MUAC, and foot length were all good predictors of small newborns, including those that are LBW, premature, or both. Although the diagnostic performance of the anthropometric measures was best when LBW was the outcome, our results indicate that any of these measures would be suitable for identifying small newborns in need of additional care. Most previous studies of newborn anthropometric measurements have documented the correlation of these measurements with LBW alone. A meta-analysis of studies that have assessed anthropometric measures as predictors of LBW found chest circumference to be the best predictor, followed by MUAC [8]. Similarly, some studies have found that chest circumference had the highest correlation (r = 0.60 to 0.85) with LBW [19–21], although others have found the correlation with LBW to be highest with MUAC, with estimates ranging from r = 0.66 to 0.95 [20–22]. Foot length has been shown in a meta-analysis to have the weakest correlation with LBW [8]. This weaker correlation between foot length and LBW was also found in a Ghanaian (AUC = 0.74, 95% CI 0.70–0.78) [19] and Indian study (r = 0.213) [20].

In the current study we determined the optimal cut-points identified for newborns who are LBW, premature, or both: for foot length we found the optimal cut points to be between 7.3 cm and 7.4 cm; for chest circumference, between 30.0 cm and 30.4 cm; and for MUAC, between 8.7 cm and 9.0 cm. Other studies have found the foot length cut-point to vary by setting: 7.2 cm, 6.3–7.85 cm and 7.4–8 cm in Europe [10], Asia [9,23–25] and Africa [26] respectively A study in Tanzania found a foot length <8 cm taken at birth was 87% sensitive and 60% specific in identifying LBW newborns, and 93% and 58% in identifying premature (<37 weeks) newborns [26]. A study in India found a foot length of <7.75 cm had 92.3% sensitivity and 86.3% specificity for identifying preterm newborns, and a foot length <7.85cm had 100% sensitivity and 95.3% specificity for identifying LBW newborns [27]. In a Ugandan study the optimal cut-point for foot length to detect small newborns was defined as 7.6 cm, with a sensitivity 85% and specificity 81% for identifying LBW newborns, and sensitivity 96% and specificity 76% for identifying premature newborns [28]. For chest circumference, most studies have recommended cut-points ranging from 29.5 cm to 33.5 cm for predicting LBW newborns [9,19,20,22,29], which are similar to the cut-points identified in in our study. For MUAC, several studies selected cut-points ranging between 9.0 cm and 10.0 cm, which were slightly higher than observed in our study [8,19–21,30]. Determining the optimal cut-point for identifying small newborns may need to be determined in individual populations as the “optimal” measurements may vary for a variety of reasons including technique, ethnic differences, different measurement tools used to determine GA, and differences in the definition of what constitutes the optimal cut-point.

As many newborns are born at home and are not visited by a health worker in the first five days of life we compared the newborns measurements within 24 h of birth with those measured on day five of life. Foot length is the least likely to be affected by newborn weight loss which often occurs in the first 10 days of life, and this weight loss is more likely to affect MUAC and chest circumference. In our study, the measurements for all three anthropometric measures were slightly lower at day five compared with the first 24 h of life. However, the estimated differences were small (<0.2 cm in all cases) and not clinically significant. In our study the average newborn’s foot length decreased in size by 0.018 cm, but the average MUAC and chest circumference decreased by 0.157 cm and 0.162 cm, respectively. In other populations, where newborn nutrition and weight gain are concerning in the first week of life, foot length may be a more robust measure and the preferred tool to screen for small newborns.

In order for interventions to be practical, methods for the identification of small newborns must also be low-cost and simple so that the tool can be implemented by minimally trained community health workers. Chest circumference, MUAC, calf and thigh circumference are more likely to be prone to inter-observer variability as landmarks to measure are less obvious. Foot length may be the more practical alternative for use in a community setting for a number of reasons: it is less likely to be affected by newborn weight loss which commonly occurs in the first 10 days of life; measuring the foot requires minimal disturbance and for the smallest of newborns, exposing a foot is unlikely to predispose to hypothermia; and it is a low cost simple tool that community health workers could use. In our study, a foot length <7.4 cm measured within 24 h of birth would have identified 85% of all LBW newborns and 80% of all pre-term newborns based on our identified cut-point. Using foot length to identify small newborns has great potential, however the reliability of its application by community workers would first need to be established [31].

We found almost half of the newborns in this study to be either LBW, premature or both. These rates are high. According to UNICEF in 2013, 16% of all newborns globally were LBW, and the rate of LBW in South Asia was 28%, with no data to estimate the prevalence from East Asia and the Pacific [32]. Although LBW data from Vietnam varies across regions and is ascertained by several different methods, the prevalence of LBW is estimated to be 8%, and is not as high as other Southeast Asian countries. The reason for the high rate of LBW and prematurity in our study requires further investigation. The high rates may be partially explained by our study being conducted in a referral hospital, with preterm births being referred from smaller health facilities; or that the study participants are ethnic minority newborns, who are known to have poorer nutritional status and have worse birth outcomes compared with their Kinh counterparts [33].

Our study had some limitations. Firstly, the determination of GA using the NBS method may have been prone to bias. The most reliable method of estimating GA is early antenatal ultrasound, however only 81% of mothers in our study had this investigation performed. Instead we used GA calculated from the NBS, which provides a less accurate assessment of GA. Nevertheless we found that GA calculated from the NBS had a strong correlation (0.9) with the GA of newborns from those women who also received an antenatal ultrasound, minimising any misclassification of prematurity. However the study paediatrician who performed the NBS was not blinded to the GA ascertained by LMP or ultrasound, nor the birth weight, and this may have influenced the individual scores given on the NBS. Secondly, almost all participants (93%) were from one ethnic minority group, the Muong, therefore the results may not be representative of newborns from all other ethnic minority groups.

## Conclusions

These results suggest that if ethnic minority Vietnamese newborns were measured within 24 h of life using the optimal cut-points provided in this report, approximately 85%, 91%, and 92% of LBW newborns and 80%, 85% and 88% of pre-term newborns would be identified using chest circumference, MUAC, and foot length, respectively. Given that the measurements taken on day five of life were on average slightly smaller than the measurements taken on the first day, if anything the numbers of LBW and premature newborns identified might be slightly over-estimated using the day five measurements, leading to a small increase in sensitivity and decrease in specificity. Nevertheless, overestimating small newborns may potentially improve neonatal outcomes overall, if community workers were also trained to provide basic post-natal care, as well as screening for small newborns. Based on these results and the relative ease of undertaking the measurement, we would recommend that foot length be used as an indicator for birthweight and GA in the community. The findings from this study are critical to identify those small newborns most at risk of dying and we have identified as a simple method which will contribute to reducing neonatal mortality.
